# Revision of the United States PharmacopœIa

**Published:** 1890-05

**Authors:** Robert Amory

**Affiliations:** P. O. Box 3281, Boston, Mass.


### REVISION OF THE UNITED STATES PHARMACOPŒIA.

A convention for the revision of the United States Pharmacopœia will be held in the City of Washington, May 7, 1890, at noon. This is a very important meeting, and one of great interest to the profession. Many faults and errors occurred in the last issue, which should be corrected in this.

For information regarding this convention, address

DR. ROBERT AMORY,
P. O. Box 3281, Boston, Mass.

